# P-230. Impact Of Fatigue As A Side Effect Of Anti-Retroviral Therapy On The Quality Of Life Of People Living With HIV: A Real-World Survey

**DOI:** 10.1093/ofid/ofaf695.452

**Published:** 2026-01-11

**Authors:** Fritha Hennessy, Abid A Kabir, Rebecca Charlton, Tim Holbrook, James Piercy

**Affiliations:** Adelphi Real World, Bollington, United Kingdom, Bollington, England, United Kingdom; Adelphi Real World, Bollington, England, United Kingdom; Adelphi Real World, Bollington, England, United Kingdom; Adelphi Real World, Bollington, United Kingdom, Bollington, England, United Kingdom; Adelphi Real World, Bollington, United Kingdom, Bollington, England, United Kingdom

## Abstract

**Background:**

Although fatigue is a known symptom for people living with HIV (PWH) and a side effect of antiretroviral therapy (ART), its burden upon PWH is not well understood. We aimed to look at the impact of fatigue on quality of life (QoL) in PWH.

**Methods:**

Data were drawn from the Adelphi Real World HIV Disease Specific Programme™, a cross-sectional survey, with retrospective data collection, of physicians and PWH in France, Germany, Italy, Spain, the United Kingdom and the United States from July 2021 – February 2024. Physicians reported demographics, clinical characteristics, fatigue as a side effect of ART and ART satisfaction for ten consecutively consulting PWH. PWH self-reported ART satisfaction, adherence via the Adelphi Adherence Questionnaire (ADAQ; scored 0–4, with 0 being perfect adherence) and QoL via PoZQoL (scored 0–5, with 5 being best QoL). Analyses were descriptive.

**Results:**

Overall, 335 physicians reported on 3373 PWH, of whom 1008 self-reported data. Mean (standard deviation; SD) patient age was 42.8 (12.8) years and 72% were cisgender male.

A total of 1913 PWH had no fatigue (NF) and 423 PWH had moderate to severe fatigue (MSF) due to ART. In total, 65% of MSF PWH were virally suppressed, 29% had no comorbidities and 32% had no side effects. For NF PWH, this was 81%, 54% and 87% of patients, respectively.

Physicians were very satisfied with current ART for 78% of NF PWH compared to only 36% of MSF PWH. A total of 79% of physicians thought NF PWH were completely adherent compared to 48% for MSF PWH.

Complete satisfaction with ART was reported by 63% of NF PWH compared to 31% of MSF PWH. Mean (SD) ADAQ scores were 0.2 (0.3) for NF PWH and 0.6 (0.6) for MSF PWH. Mean (SD) PozQoL scores were 3.6 (0.7) for NF PWH and 2.6 (0.7) for MSF PWH.

**Conclusion:**

MSF was associated with low physician and PWH satisfaction with current ART, as well as low adherence and QoL scores. MSF PWH also commonly experienced comorbidities and treatment side effects, with fewer than half of PWH fully adherent to their treatment. Fatigue should be addressed as a prominent clinical burden in the treatment of PWH, with future research elucidating the link between fatigue severity and ART type.

**Disclosures:**

All Authors: No reported disclosures

